# Sex Differences in Money Pathology in the General Population

**DOI:** 10.1007/s11205-014-0756-x

**Published:** 2014-09-11

**Authors:** Adrian Furnham, Sophie von Stumm, Mark Fenton-O’Creevy

**Affiliations:** 1 Psychology, University College London, London, UK; 2 Norwegian Business School, Oslo, Norway; 3 Department of Psychology, Goldsmiths University of London, London, UK; 4 The Open University Business School, The Open University, Walton Hall, Milton Keynes, MK7 6AA UK

**Keywords:** Money, Sex, Pathology, Gender

## Abstract

This study examined sex differences in money beliefs and behaviours. Over 100,000 British participants completed two measures online, one of which assessed “money pathology” (Forman in Mind over money, Doubleday, Toronto, 1987), and the other four “money types”, based on the emotional associations of money (Furnham et al. in Personal Individ Differ, 52:707–711, 2012). Nearly all measures showed significant sex differences with medium to large effect sizes, and with females exhibiting more “money pathology” than males. The biggest difference on the money types was on money being associated with generosity (money representing love) where men scored much lower than females, and autonomy (money representing freedom) where men scored higher than women. For men, more than women, money represented Power and Security. Men were more likely to be Hoarders while women did more emotional regulatory purchasing. Implications and limitations of this study are discussed.

## Introduction

This study is concerned with sex differences in attitudes to, beliefs about, and the uses of money. It concentrates on sex differences in money pathology, which is a topic that has recently received more empirical attention.

There is a growing, literature on the psychology of money (Baker and Jimerson 1992; Belk and Wallendorf 1990; Crawford 1994; Ealy and Lesk 1999: Furnham and Argyle 1998; Norvelitis et al. 2006; Lea and Webley 2006; Tang 2007, 2010; Wiseman 1974) with various recent studies attesting to its psychological power (Rick et al. 2008; Vohs et al. 2006). Further, the rise of behavioural economics has led to the many popular books on the topic (Belsky and Gilovich 1999). Recent interest has been particularly concerned with the relationship of money with happiness (Cone and Gilovich 2010), the role of money in personal relationships (Dakin and Wampler 2008; Shapiro 2007) as well as the role of money in the work place (Tang et al. 2006).

Some clinicians, particularly those with a background in psychoanalysis, have written about disordered money beliefs and behaviours (Forman 1987; Goldberg and Lewis 1978; Rendom and Krauz 1992; Mellan 1994; Wilson 1999). Some researchers have developed typologies identifying specific emotional meanings that people attach to money. Goldberg and Lewis (1978) have suggested that money has essentially four emotion connotations: money represents *security* (a primary way of staving off anxiety), *power* (method to gain importance dominance and control), *love* (a manifestation of, and substitute for affection) and *freedom* (a necessity to acquire what you want). Many other researchers have come up with similar typologies (Furnham 2014). Nearly all this work is based on case studies of middle-class people. Others have attempted to develop psychometrically valid instruments that specifically measure their dysfunctional beliefs and behaviours (Furnham et al. 2012; Klontz et al. 2012).

Many clinicians have referred to “money insanity” of their clients: that is, the highly irrational behaviour of some people with respect to their use of money. This has also been called “money neuroses” and characterised by extreme money worries of many kinds (Forman 1987). Further, there is a recent interest in “money genograms” which are social and emotional values attached to money originating in childhood family relations (Mumford and Weeks 2003). Psychotherapists have argued that money beliefs and behaviours are not isolated psychic phenomena but integral to the person as a whole and measure of underlying pathology (Forman 1987; Matthews 1991). Thus, people who withhold money may have tendencies to withhold praise, affection or information from others (Goldberg and Lewis 1978). The fact that it is imbued with so much meaning may account for its framing power (Vohs et al. 2006).

Forman (1987) noted that of all the neuroses, the “money neurosis” is most widespread. Like all neurotic processes it involves unresolved conflict associated with fear and anxiety that may relate directly to maladaptive, self-defeating, irrational money behaviour. Forman argued that too many people have a simple equation like money equals love in the sense that the amount of money you spend on a person is proportional to the amount of love you have for them. To help people identify their neurosis, Forman (1987) developed a general *money sanity* scale, which is used in the current study and whose psychometric properties have been previously evaluate (Furnham 1996).

Many popular writers have attempted to differentiate “money types”. Thus Mellan (1994) described nine; hoarder, spender, binger, monk, avoider, amasser, worrier, risk taker, and risk avoider. The four most commonly occurring money-associated emotions have been identified by different authors: Security, Power, Love and Freedom (Furnham and Argyle 1998; Furnham et al. 2012; Goldberg and Lewis 1978; Mumford and Weeks 2003). Some of these measures have been used to demonstrate, for instance, that money attitudes and financial capabilities each contribute independently to the risk of experiencing adverse financial outcomes (von Stumm et al. 2013).

Some books on money have been written exclusively for women (Ealy and Lesk 1999; Orman 2007; Pine 2009; Webb 2008) but few provide data on sex differences. There is even an online magazine on the topic (http://womenmoneyandsuccessmag.com/). There is also a literature on money in relationships (Burgoyne 2004; Elizabeth 2001; Nyman 2003; Pahl 1995) suggesting it can be a major cause of friction because of what it represents. A sociological perspective on sex differences in money behaviours stresses structural or societal causes like the fact that women earn less money than men; that they often experience longer periods of financial dependence and that money is structurally organised through the institutions of work and family along gendered lines (Furnham and Wilson 2011).

Many clinicians speak of the two different “gender cultures” with respect to money as a result of socialisation (Mellan 1994). Men are more competitive with money associating it with freedom and power while women feel more needy and vulnerable and therefore associate it with security and love (Mumford and Weeks 2003). There are some small scale studies and reviews that suggest there are significant sex differences in various money-related pathologies like impulse purchasing, compulsive buying, hoarding and credit card debt (Cheng and Furnham 2014). There is also evidence women have less objective financial knowledge and different financial planning behaviours than men (Woodyard and Robb 2012). However, there are few large scale empirical studies to show support for these sex difference hypotheses.

There have been two large scale surveys in this area but both may now be out-of-date. In an early American survey of over 20,000 adults Rubinstein (1981) found that males were found to be more confident and self-assured about money than the women, being more highly content about their financial situation, as well as feeling more in control of it, and predicting a higher earning potential for themselves. No statistical analysis of this data was provided. Later, Lynn (1991a, b) also investigated sex differences in money beliefs on a very short four item scale in groups of around 300 students from 20 countries and showed a general trend for males to attach more value to money than females. He proposed that males generally tend to be more competitive and define their identity via money because they are traditionally the bread winners; women identify with home making and children (i.e. sex differences in socialisation).

Furnham and Okamura (1999) in a small scale British study found that females appeared to be less inclined to take moral risks for money, as well as being less inclined to believe that significant money was only acquired through chance. Females were, however, found to hold no more negative emotions about money than men, despite being more prone to (psychosomatic) depression overall.

Despite the fact that males attach more value to money than females, Wang (2009) found that male investors exhibited stronger risk-taking behaviour than female investors. This was attributed to the fact that male investors tended to have more knowledge than female investors.

The present study used a large population sample to investigate sex differences in money beliefs and behaviours. Previous studies using these measures had small samples (N = 409, Furnham et al 2012; N = 277, Furnham 1996). Further, it was possible to investigate the psychometric properties of these measures paying particular attention to their factor structure. From previous studies as well psychotherapeutic and sociological speculations it was hypothesised:

Hypothesis 1: Females will score higher on overall money pathology, and associate money more with Love (Hypothesis 2) and less with Power (Hypothesis 3) and Freedom/autonomy (Hypothesis 4) than males.

## Methods

### Participants

There were 109,472 participants of which 46.7 % were male. Their mean age was 39.58 years (SD = 14.35). Most were “White British” (90.3 %). In all, 23 % had, as their highest qualification GCSE/O Levels/ 10th Grade, while 20 % had A Levels (12th Grade) and 37 % were graduates. As regards to their annual gross income 7.3 % earned less than £9,999, 22.7 % between £10,000 and 19,999, 17.4 % £20,000 and £29,999, 14.8 % between £30,000 and £39,999, 12.1 % between £40,000 and £49,999, 15.3 % over £50,000 and 6.6 % said either they did not know or preferred not to say. In all, 61.7 % were in full or part-time employment, 8.4 % homemakers and 3 % unemployed. Asked how many people were financially dependent on them 54.9 % said none; 19.3 % one, 14.4 % two. Asked to self-define their social class 54.0 % said working (lower 6.6 %, middle 35.0 %, upper 12.3 %) and the remainder middle class (lower 16.9 %, middle 24.4 %, upper 4.4 %).

### Measures

*The Money Sanity Questionnaire* (Forman 1987): This is a 20 item questionnaire that measures a person’s money sanity or the extent to which their attitudes to, and use of money is problematic. It has a dichotomous “yes”/”no” format which is not ideal and some questions have shown floor and ceiling effects (Furnham 1996). It was based on extensive clinical work as well as reviews of the area. It has previously been used in other studies in the area (Furnham and Okamura 1999). High scores imply “insanity” or the presence of maladaptive money beliefs and behaviours. In the present study the alpha of the total scale was .67, with females scoring higher than males (Mean 5.36, SD = 3.07 vs 4.82, SD = 2.92; Cohen’s d = .18).

*Money Attitudes Scale* (Furnham et al. 2012). This 16-item questionnaire assesses attitudes to money, rated on a Likert scale ranging from 1 (strongly disagree) to 5 (strongly agree). Factor analytic work provided evidence of four factors but not exactly as the authors expected but they retained the labels: security, freedom, power and love.

### Procedure

Participants took part on an on-line BBC survey on money attitudes, beliefs and behaviours knowledge as part of 10 planned scientific investigations. It was part of a ten part science series and the authors were asked to take part because of their knowledge of the subject. The aim of the BBC was to bring social science alive and let an audience participate. Participants remained anonymous and responded within 6 weeks of the survey going live. A program was written such that they got individual feedback on their responses. The UCL research ethics committee reviewed and accepted the proposal.

### Statistical Analysis

In a first step, the tetrachoric correlation matrix of the money sanity items was subjected to unit-weighted least squares exploratory factor analysis (EFA) in a subsample. (Tetrachoric correlations were used because item responses are dichotomous). The number of retained factors was identified according to the Kaiser Normalization rule and parallel analysis. Factor solutions were promax rotated. Subsequently, the obtained factor solution was confirmed using structural equation modeling (again using tetrachoric correlations and robust estimators) in two further subsamples, and then fitted to the overall sample. For each factor, unit-weighted composites were computed. In a second step, the Money Attitudes Scale (Furnham et al. 2012) was subjected to factor analysis with oblimin rotation, following Kline’s (1986) recommendations, in a random subsample. Factor identification was initially based on the examination of the scree plot and the Kaiser-rule of Eigenvalues above one, and then confirmed with parallel analysis. The obtained factor solution was subsequently tested in two further subsamples. In a third step, correlations (and partial correlations controlling for income) between money sanity and money attitudes were computed. Finally, sex differences in the study variables were tested. Because of the current study’s large sample size, making even very small differences significant, we report effect sizes (Cohen’s d: differences expressed in pooled standard deviation units) rather than *p* values.

## Results

Two random samples were drawn using the SPSS algorithm for selecting a proportion of the sample (2.5 % in our case) at random. The first EFA recommended retaining five factors in a first random subsample (*N* = 3,223), based on the Kaiser Eigen-value criterion but showed a negative residual variance for one item, which was confirmed by a second EFA in a second random subsample (*N* = 3,228). The item was 8:“If I had enough money, I would never work again”. The EFA was re-run excluding the negative residual variance item in both subsamples, each yielding admissible five-factor solutions. Parallel analyses, however, suggested retaining only four factors in both subsamples. In line with this, a series of CFAs (using a structural equation model) in a third subsample (N = 44, 569) showed better fit index values for four factor solutions. Across samples, the four factor solution was characterized by four items showing no salient loadings on any factor, two items cross-loading on two factors, and one item showing considerably variabilities in factor loadings. Table 1 shows the loadings obtained in a four-factor CFA based on the overall sample. The model fit was adequate (RMSEA = .064; CFI = .887). With the exception of one item (fourth factor; 8, see above), all items had salient loadings on their respective factors. Two items were specified to cross-load on factors 1 and 2, in line with the results of the EFA. The first factor was identifiable as worried spending, while the second factor seemed to describe spenders in denial. The third factor appears to describe individuals who compensate for negative emotions by spending money, and the fourth factor included hoarding and saving behaviors. That said, the fourth factor also included an item on manipulating people with money and had poorer internal consistency. Unit-weighted composite scores were calculated for each factor.

**Table 1:** Frequencies for money sanity items, factor loadings and factor descriptives

Items	Yes (%)	1	2	3	4
Do you resent having to pay full price for any item when you shop?	51.5				.67
Do you use money to control and manipulate others?	4.5				.46
Would you walk out of your way to save a bus fare you could easily afford?	35.3				.43
Do you hold onto, or hoard your money?	35.2				*.16*
Do you buy things when you feel anxious, bored, upset, depressed or angry?	33.7			.79	
Do you buy things you don’t really need because they are great bargains?	33.1			.69	
Do you spend a large proportion of your free time shopping?	12.1			.68	
Are you reluctant to learn about practical money matters?	9.4		.80		
Do you refuse to take money seriously?	9.1		.67		
Do you regularly exceed the spending limit on your credit card?	4.7	.43	.50		
Are you constantly puzzled about where your money goes or why there is none left at the end of each month?	23.0	.47	.49		
Do you find yourself worrying about the spending, using or giving of money all the time?	4.8	.88			
Are you increasingly anxious about whether you can pay your bills each month?	18.5	.83			
Do you think about your finances all the time?	33.0	.77			
Do you lie awake at night trying to figure out a way to spend less money and save more, even though you are already saving money?	21.9	.73			
When you ask for money are you flooded with guilt or anxiety?	45.0	.57			
Are you inhibited about talking to others about money, particularly about income?	38.1				
Does gambling make you feel a burst of excitement?	21.3				
Do you often gamble and spend large sums on your bets?	2.2				
Do you spend money on others but have problems spending money on yourself?	39.0				
Factor 1		–			
Factor 2		.30	–		
Factor 3		.35	.55	–	
Factor 4		.35	−.07	.10	–
**Alpha**		**.74**	**.52**	**.55**	**.64**
**Mean**		**1.87**	**0.46**	**0.79**	**1.27**
**SD**		**1.84**	**0.80**	**0.93**	**1.00**

Factor loadings according to confirmatory factor analysis are shown. Loadings below .30 are shown in italics

In a first random subsample (N = 3,242) scree plot and Eigenvalues of the factor analysis of the money attitude scale suggested the extraction of four factors; the parallel analysis supported this solution. After promax rotation, one item did not load substantially on any factor, and it was therefore excluded from any further analysis. This initial factor solution was confirmed in a second subsample using structural equation modeling (N = 3,251), and then was fitted to the overall sample. Table 2 shows the factor loadings of the retained items on their respective factors. The solution accounted for 53.7 % of the total variance (N = 109,472). The factors were readily interpretable and closely matched Furnham et al. (2012) findings, including a power oriented money attitude; a security focused attitude; an affection or generosity by money attitude; and an autonomy worshipping attitude. Factor regression scores were computed and saved to represent each attitude factor; their coefficient alpha values were .75 for power; .61 for security; .64 for generosity; and .63 for autonomy.

**Table 2:** Results from the meaning on money questionnaire

	1	2	3	4
Money is important because it shows how successful and powerful you are	.82			
The best thing about money is that it means you can influence others	.67			
I have always been inspired by powerful tycoons who make lots of money	.59			
Money can help you be accepted by others	.46			
The best present you can give to someone is money	.45			
You can never have enough money	.44			
I’d rather save money than spend it		.75		
It is important to have savings, you never know when you may urgently need the money		.53		
Relative to my income I tend to save quite a lot of money		.53		
If I don’t save enough money every month I get very anxious		.39		
I often demonstrate my love to people by buying them things			.77	
I am very generous with the people love			.62	
With enough money, you can do whatever you want				.84
The main point of earning money is to feel free and be free				.50
There are very few things money can’t buy				.41
Alpha	.75	.61	.64	.63

Loadings below .40 are omitted

Coefficient alpha values were .75 for power; .61 for security; .64 for generosity; and .63 for autonomy

Table 3 shows the correlations between money sanity and money attitudes. The power money attitude was positively, albeit weakly associated with all money sanity factors. Security shared the greatest amount of variance with denial spending (negative, r = −.29) and hoarding (positive, r = .38), in line with the theoretical rationales of these three constructs. Generosity was most strongly, positively associated with compensation spending behaviors (r = .28), while autonomy was most substantially correlated with worried spending (r = .20). Overall, money attitudes and money sanity factors were meaningfully empirically associated.

**Table 3:** Correlations between money attitude and money sanity factors (N = 109, 472)

		1	2	3	4	5	6	7
1	Power							
2	Security	.09						
3	Generosity	.34	−.08					
4	Autonomy	.55	.11	.22				
5	Worried spending	.21	−.06	.17	.20			
6	Denial spending	.17	−.29	.17	.13	.53		
7	Compensation	.17	−.23	.28	.12	.26	.31	
8	Hoarder	.17	.38	−.04	.12	.10	−.06	−.02

Because of the size of the sample all correlations are significant

Table 4 shows the means, Standard Deviations and Cohen’s d for the sex differences in money variables. Men were more Power, Autonomy and Security oriented with regard to money than women. Conversely women were much more Generous with their money. They also scored higher on Worried, Compensatory and Denial spending than their male counterparts, who exceeded in Hoarding. Overall, men appear to associate money with Power, Security and Autonomy and were more likely to Hoard. By contrast, women share their money and score higher on problematic spending habits.

**Table 4:** Sex differences (Cohen’s d) in money attitudes and sanity factors

	Male		Female		*d*
	Mean	SD	Mean	SD	
Power	0.15	0.94	−0.13	0.85	0.32
Security	0.09	0.83	−0.08	0.84	0.20
Generosity	−0.06	0.82	0.05	0.86	−0.69
Autonomy	0.05	0.88	−0.04	0.86	0.55
Worried spending	1.65	1.72	2.06	1.92	−0.23
Denial spending	0.43	0.78	0.49	0.81	−0.08
Compensation	0.56	0.79	0.99	1.00	−0.48
Hoarder	1.40	1.02	1.15	0.97	0.25

## Discussion

This paper set out to investigate sex difference in money pathology using two different self-report measures. All hypotheses were confirmed. Both measures showed many significant sex differences at the item, subscale and total scale level and effect sizes for the internally reliable scale were modest to large. Compared to males, females were *Worried Spenders*; they shopped as a form of therapy; and they worried more about money. Overall, females exhibited a higher “Money Insanity” score (Forman 1987; Cheng and Furnham 2013).

Whilst some items on the 20 item money insanity scale showed floor effects (around 5 % or less of the sample agreed (i.e. said “yes” to the item) some suggested that relatively large numbers endorse this money madness. Thus over 40 % said they worried about spending money all the time (item 1) and half agreed that they resent paying the full price in shops. Nearly half said they were flooded with guilt and anxiety when asking for money and over a third said they felt anxious in spending money on themselves. A third said they thought about money all the time. However the factor analysis of this measure gave a reasonably interpretable output. Furthermore the dimensions do reflect the specific money “pathologies” identified by various different researchers working in the area (Goldberg and Lewis 1978; Matthews 1991).

There are three issues with the money “pathology” scale that merits comment. First, it is possible a certain amount of dissimulation occurred despite the fact that the questionnaire was anonymous. Thus, very few agreed the possibly unacceptable idea that they use money to control or manipulate others. Second, it is possible to argue that some of these beliefs and behaviours labeled “pathological” or manifestations of “insanity” are both normal and healthy. Third, it may be that some interesting and important “pathologies” such as compulsive saving or hoarding are missing. Nevertheless the scale gives a picture of many people being obsessed by money in that they think about it the whole time and of money being a powerful source of mainly negative emotions.

The two typologies were related in ways which make intuitive sense (see Table 2). Thus those who associated money with security tended to be hoarders and those who associated money with generosity tended to use money emotionally and compensatorially. It seems that whichever of the various scales that exist that attempt to measure attitudes to money (Furnham and Argyle 1998) similar dimensions arise like Spender, Saver/hoarder, Anxious Worrier, or Spendthrift and Tightwad (Rick et al. 2008).

The most important data for this study are in Table 4. All the sex differences were highly significant: eight sex effects one could be considered small, four medium and three large. The biggest effect was for generosity. This has been labeled in different studies as Affection or the association of money with Love. It refers to giving and buying of presents as tokens of love, esteem and friendship. The score for women is much higher than for men which confirms previous clinical and empirical data (Furnham et al. 2012: Goldberg & Lewis 1978). This finding is complimented by the analysis which showed the second biggest effect size to be ‘compensation’: shopping behavior which relates to shopping for emotional regulation purposes, sometimes known as “retail therapy”.

Two other large effect size differences also confirm previous literature namely the association of money with Power and Autonomy. For men, more than for women, money represents freedom and the ability to achieve goals. Both scales did however correlate significantly with the money pathology scales, particularly worried spending (see Table 2), suggesting that these associations also could be conceived of as “pathological”.

This was a cross sectional study of a large European population group measured at a time of economic difficulty. Socio-economic and cultural factors could have influenced these results As such it could not answer questions of how these sex differences arise; that is to what extent they have a biological or evolutionary basis or are a product of socialization and social structure or both. There has recently been an interest in the evolutionary psychology of consumption where socio-biological explanations are offered for numerous sex differences in shopping, spending and responding to advertising (Hantula 2003; Saad and Gill 2001). On the other hand it has been argued that sex differences in money beliefs and behaviours are essentially a product of structural institutions and socialization and can therefore be relearned (Matthews 1991).

Whereas the study had a large, reasonably population there were acknowledged limitations with the measures used. There are now more and psychometrically improved questionnaires for use in this area (see Furnham 2014, for review). What all studies in this area would benefit from most is longitudinal studies of actual money beliefs and behaviours to ascertain their causal structure measuring and exploring the interaction of economic, psychological and sociological variables (Cheng and Furnham 2013; Furnham and Cheng 2012).
